# Testosterone propionate and *Swarna Bhasma* treatment modulated D-galactose induced reproductive alterations in male Wistar rats: An experimental study

**DOI:** 10.18502/ijrm.v21i4.13270

**Published:** 2023-05-08

**Authors:** Aashish Kumar Netam, Vikas Pankaj Bhargava, Rambir Singh, Poonam Sharma

**Affiliations:** ^1^Department of Zoology, Indira Gandhi National Tribal University, Amarkantak, Madhya Pradesh, India.; ^2^Department of Horticulture, Aromatic and Medicinal Plants, Mizoram University, Aizawl, Mizoram, India.

**Keywords:** D-galactose, Aging, Testosterone propionate, Swarna Bhasma, Testis, Hypogonadism, infertility.

## Abstract

**Background:**

The male reproductive system undergoes several adverse age-related changes like decreased hormone synthesis, sperm count, and testicular alteration that can impact on fertility.

**Objective:**

The study aims to investigate the effects of testosterone propionate (TP), and ayurvedic formulation *Swarna Bhasma* (SB) on D-galactose (D-gal) induced reproductive aging in male Wistar rats.

**Materials and Methods:**

60 male Wistar rats were divided into 10 groups of 6 animals. Reproductive aging was induced by D-gal (150 mg/kg Bwt) exposure for 60 days. The rats were then treated by post and combination treatment with TP (2 mg/kg Bwt) and SB (6.75 mg/kg Bwt). Then sperm parameters, reproductive hormones, inflammatory markers, testicular antioxidant enzymes, steroidogenic enzymes, and histological manifestation of testis were evaluated.

**Results:**

Exposure of D-gal caused significant (p < 0.001) decrease in serum testosterone (T), testicular steroidogenic, and antioxidant enzymes. Administration of TP increased the serum T level, testicular antioxidant enzymes, and spermatogenic profile at a significant level of (p < 0.001) compared to D-gal. Further, the SB treatment significantly (p < 0.001) elevated the serum T level, sperm count, testicular antioxidant enzymes, steroidogenic enzymes, when compared to D-gal.

**Conclusion:**

Both the treatment of TP and SB treatments recovered the reproductive impairments caused by D-gal. However, exogenous T supplementation via TP administration is associated with various side effects during long-term use. SB is an Ayurvedic formulation having a long history of usage in India. The current findings suggest that the SB may be used as a good alternative for potentiating reproductive function in aging males.

## 1. Introduction

Aging is a time-dependent gradual progressive deterioration marked by a deceleration of various physiological functions, leading to increased vulnerability and mortality (1). The reproductive system is one of the first biological systems to show age-related changes with reduced fertility in both women and men. Aging related changes in male reproductive system primarily occurs in testis with increased testicular apoptosis, low-sperm count and decreased secretion of sex hormones ultimately leading to infertility (2, 3). D-galactose (D-gal) is a widely used xenobiotic for the experimental induction of aging in laboratory animals (4). Recently, the D-gal at a dose ranging from (100-200 mg/kg) body weight (Bwt) for 6-8 wk has been optimized for investigating male reproductive aging in rodent models. Male reproductive aging has been characterized by decreased testosterone (T) production, altered gonadotropic hormone release, a lower sperm count, increased sperm abnormalities, and testicular degeneration (4, 5).

T is a primary male sex hormone that plays an important role in the body by regulating sex drive (libido), sperm production, bone mass, muscle mass, and strength (6, 7). As men age, there is a slow and continuous decrease in T levels, leading to a condition called hypogonadism (gonadal failure and hypothalamic-pituitary axis failure) (8). The decline level of T is responsible for age-related impairment in spermatogenesis leading to infertility (9, 10). The treatment of hypogonadism or lower androgen concentration consists of testosterone supplementation therapy (TST) to normalize serum T levels (11, 12). Testosterone propionate (TP) is a fast-acting testosterone ester injectable compound mainly used as TST agent for the treatment of low-testosterone level in men. Recent studies have suggested that T therapy improves libido, sexual activity, erectile function, and fertility (13). According to some researchers, administering exogenous T in various forms of TST may be harmful to the liver, reduce insulin's effect on lipid metabolism, and increase the risk of cardiovascular disease, stroke, and prostate cancer (14-16). Therefore, an alternative to TST is required, preferably one that increases endogenous T production to normalize steroidogenic function in aging human male (17).

The use of natural products in potentiating the male reproductive system has been associated with human civilization since antiquity (18). *Swarna Bhasma* (SB), also known as gold ash, is a metallic formulation in traditional *Ayurvedic *medicine that contains nano and colloidal gold particles. SB is widely known as a metabolic booster and ayurvedic physicians used to treat different diseases such as tuberculosis, cancer, bronchial asthma, rheumatoid arthritis, diabetes mellitus, anemia, nervous, and reproductive system related disorders (19-21). *Ayurveda* claimed that SB improves sperm count, quality and quantity of semen, and sexual activity (20, 22).

However, to our knowledge, there are no experimental reports available on reproductive system enhancement activity of SB in aging animals/human beings. Hence, the present study was planned to study the effect of SBon various reproductive parameters in D-gal induced aging male Wistar rats, and TP was used as a standard drug.

## 2. Materials and Methods 

### Animals

60 male Wistar albino rats (120-150 gr, 1-2-month-old) were obtained from the Central Drug Research Institute, Lucknow, Uttar Pradesh, India. Experimental animals were acclimatized for 15 days in the Institutional Animal House of Indira Gandhi National Tribal University, Amarkantak, Madhya Pradesh, India. The animals were given food and water ad libitum and were kept in an environment with ambient temperatures of 25 ± 3 C, relative humidity of 60 ± 5%, and a 12-hr light/dark cycle.

### Chemicals

The D-gal was purchased from Himedia Pvt. Ltd., India, whereas the TP and sesame oil used in the current study were procured from Sigma-Aldrich, US. A high-graded SB (Batch number 47), honey, and trikatu were acquired from Shree Baidyanath Ayurved Bhawan Pvt. Ltd., Jhansi, India.

### Experimental schedule 

A total of 60 rats were randomly divided into 10 groups of 6 animals based on their Bwt. The dose of D-gal and TP were selected based on earlier studies (1, 5, 23). Further, the dose of SB for the current study was calculated from the therapeutic dose of SB for human, using dose conversion factor from human to rats (24). D-gal and TP were administered daily by subcutaneous injection, whereas SB was administered orally by gavaging daily for 60 days. The normal saline, trikatu in honey and water, and sesame oil were used as a vehicle for administering D-gal, SB, and TP, respectively. The detailed experimental schedule is given in (Table I)*.*

The behavioral and Bwt changes were monitored throughout the experiment. At the end of the experiment, the animals were humanely sacrificed by halothane inhalation. The testis and other accessory reproductive organs (epididymis, prostate, and seminal vesicle) were quickly dissected and washed with ice-cold 0.9% normal saline. The organs were weighed and transferred to -70 C for further study. Blood samples were obtained by cardiac puncture, and serum was isolated and stored at - 20 C till further analysis.

### Sperm parameters

The epididymal sperm count assessment was done using the hemocytometer method with certain modifications (25). Briefly, the outer covering of cauda epididymis was removed, minced, and homogenized in 1 ml of 0.9% NaCl and 0.05% triton-X solution. The resultant sperm suspension is then diluted 10 times and centrifuged at 8000 RPM for 2 min. Homogenate (10 µl) was placed in the red blood cell chamber of hemocytometer and sperms were counted at 400 × magnification.

For the sperm abnormalities assessment, a piece of the cauda epididymis was minced, 1 ml of 0.9% saline and 1 ml of 10% neutral buffer saline were added. For the assay, the above suspension was further diluted with water, and 1 ml of eosin stain (1%) was added to it. The above solution was further incubated at room temperature for 1 hr. The sperm smear was prepared by pouring a drop of the suspension on the slide and examined at 400 × magnification using a trinocular light microscope (Olympus Microscopes, Tokyo, Japan). On each slide, 200 sperm were analyzed for various head and tail abnormalities, and results were expressed as percentage abnormalities. In order to measure sperm motility, the distal end of the epididymis was removed and minced in 2 ml of Dulbecco's phosphate buffer saline and was kept at 36-38 C for further analysis. The minced cauda was placed in a water bath and kept for 1-5 min to disperse the sperms. The sperm suspension (5-10 µl) was placed into the WBC counting chamber, and the number of motile sperms were counted. The hemocytometer was then placed at 40-50 C for 1 min to kill the sperm, and the total number of dead sperms were counted. The results were presented as percentage motility (26).

### Hormone assay

The serum T level, luteinizing hormones (LH) and follicle-stimulating hormone (FSH), were measured using rat-specific enzyme-linked immunosorbent assay (ELISA) Kits (Elabscience, China). The procedure for the assay was followed according to the manufacturer's instructions.

### Steroidogenic enzymes 

Testicular 3-beta-hydroxysteroid dehydrogenase (3β-HSD) and 17-beta-hydroxysteroid dehydrogenase (17β-HSD), were assayed by rat-specific ELISA kits (Cusa Bio, China) as per the kit manufacturer's instructions.

### Serum inflammatory markers

The serum samples were processed to assess the various inflammatory markers nuclear factor kappa-light-chain-enhancer of activated B cells, tumor necrosis factor (TNF-α), interleukin-1beta (IL-1β), and interleukin-6 (IL-6), using rat-specific ELISA kits (Elabscience, China), as per the kit manufacturer's instructions.

### Antioxidant enzyme assay 

A 10% (w/v) phosphate buffer (pH 7.4 + 150 mM KCl) ice-cold solution was used to homogenise the testis. The lipid peroxidation (LPO) and reduced glutathione (GSH) were determined from one part of the homogenate. Another part of the homogenate was centrifuged at 9000 RPM to obtain the supernatant (S9) fraction. The S9 fraction was used to calculate total protein, superoxide dismutase (SOD), catalase (CAT), glutathione reductase (GR), glutathione peroxidase (GPx), and glutathione-S-transferase (GST). The LPO and GSH level in the testis were estimated by the previously used methods (27, 28). The SOD and CAT level were measured using earlier described procedures (29, 30). GPx, GST, and GR were assessed as per the previous protocols (30, 31). Lowry method was used for total protein estimation (32).

### *StAR* gene expression study

Total RNA from the part of the testis was extracted using the Aurum ${}^{\text{TM}}$ Total RNA Mini kit (Bio-Rad, USA) according to the manufacturer's instructions. The RNA purity and concentration were measured by spectrophotometric analysis at A ${}_{260}$ /A ${}_{280}{}_{}$ nm absorbance ratio. The cDNA was prepared from extracted mRNA using the iScripts ${}^{\text{TM}}$ cDNA synthesis kit (Bio-Rad, USA).

cDNA was amplified by quantitative real-time PCR (Bio-Rad, USA) using SsoFast ${}^{\text{TM}}$ EvaGreenⓇ Supermix (Bio-Rad, USA). Rat-specific primers were designed for the genes of interest: StAR (Steroidogenic acute regulatory protein), and RPL-19 (Ribosomal protein L-19); details of the primers used are listed in (Table II). Housekeeping gene *RPL-19* was used as a reference gene. Cycling stages were followed as: - Step 1- 3 min at 95 C; step 2- 40 cycles at 95 C for 10 sec, 60 C for 30 sec, and 30 sec at 72 C; step 3- dissociation stage. The threshold cycle values of each sample were used for PCR data analysis. Relative quantitative RT-PCR gene expression was analyzed using the fold change method by calculating 2 ${}^{-Ct}$ .

### Histopathological examination 

The testes were stored in 10% formalin. A transverse section (5-µm thick) of testis was cut with a semiautomatic rotary microtome (Yarco YSI 060, US) and stained with hematoxylin-eosin (H&E). The section was examined under a microscope at 400x magnification.

**Table 1:** Experimental schedule


**Healthy control (C)**	**Normal diet and water ** ***ad libitum*** ** for 60 days**
**VC-I**	Sesame oil (100 μl/kg Bwt) for 60 days
**VC-II**	Trikatu (50 mg/kg Bwt) and Honey: Water (2:3 ratio) for 60 days
**Exposure (D-gal)**	D-gal (150 mg/kg Bwt) exposure for 60 days
**Post treatment-TP (Post-TP)**	D-gal (150 mg/kg Bwt) exposure for 60 days followed by treatment of TP (2 mg/kg Bwt) for another 60 days
**Combination-TP (Comb-TP)**	Co-treatment of D-gal (150 mg/kg Bwt) and TP (2 mg/kg Bwt) for 60 days
**Treatment-TP (T-TP)**	TP (2 mg/kg Bwt) treatment for 60 days
**Post treatment-SB (Post-SB)**	D-gal (150 mg/kg Bwt) exposure for 60 days followed by treatment of SB (6.75 mg/kg Bwt) for another 60 days
**Combination-SB (Comb-SB) **	Co-treatment of D-gal (150 mg/kg Bwt) and SB (6.75 mg/kg Bwt) for 60 days
**Treatment-SB (T-SB) **	SB (6.75 mg/kg Bwt) treatment for 60 days
VC: Vehicle control, Bwt: Body weight, D-gal: D-galactose, SB: *Swarna Bhasma*, TP: Testosterone propionate	

**Table 2:** Primer sequence


**Primer**	**Sequence**	**Reference No.**
	Forward- 5 ' -CATCCAGCAAGGAGAGGAAG-3 '	67183557
**StAR**	Reverse- 5 ' -CACCTGGCACCACCTTACTT-3 '	67183558
		Forward- 5 ' -CGTCCTCCGCTGTGGTAAA-3 '	437926
**RPL-19**	Reverse- 5 ' -AGTACCCTTCCTCTTCCCTATGC-3 '	437927
	StAR: Steroidogenic acute regulatory protein, RPL-19: Ribosomal protein L-19		

### Ethical considerations

The present research was approved by the Institutional Animal Ethical Committee of Indira Gandhi National Tribal University, Amarkantak, Madhya Pradesh, India with approval number IGNTU/IAEC/2018/10. The animals were cared for and treated as per the rules prescribed by The Committee for Control and Supervision of Experiments on Animals (CPCSEA), Govt. of India.

### Statistical analysis

Statistical analysis was performed by ANOVA and post hoc least significant difference tests using GraphPad Prism software version 8 (GraphPad Software, USA). Data were expressed as mean ± standard error (SE). The results were expressed as statistically significant at a probability level of p < 0.05.

## 3. Results 

In context to all the parameters, no significant difference was observed between the healthy control group and the vehicle control groups (VC-I, VC-II). Hence, healthy control was used for all comparisons.

### Body and organ weight

The Bwt and reproductive organs of animals of the D-gal group were significantly decreased when compared to the control group. The treatment of TP and SB substantially increased the Bwt and reproductive organs weights decreased by D-gal. The results of Bwt and reproductive organs weight has been summarized in the (Table III).

### Sperm parameters

The results showed a significant (p < 0.001) decrease in the epididymal sperm counts in the D-gal administered groups as compared to the control groups. The injection of TP and oral administration of SB significantly elevated the reduced sperm count in the Post-TP (p = 0.73), Comb-TP (p = 0.12), Post-SB (p = 0.39), and Comb-SB (p < 0.01) groups when compared to the exposure group (D-gal).

A significant (p < 0.001) decrease in the mean sperm motility was observed in the D-gal group when compared to the control group. On TP and SB administration, a non-significant (p > 0.05) increase in sperm motility was seen in Post-TP, Comb-TP, Post-SB, and Comb-SB groups when compared to the exposure group (D-gal) (Table IV).

**Figure 1:**
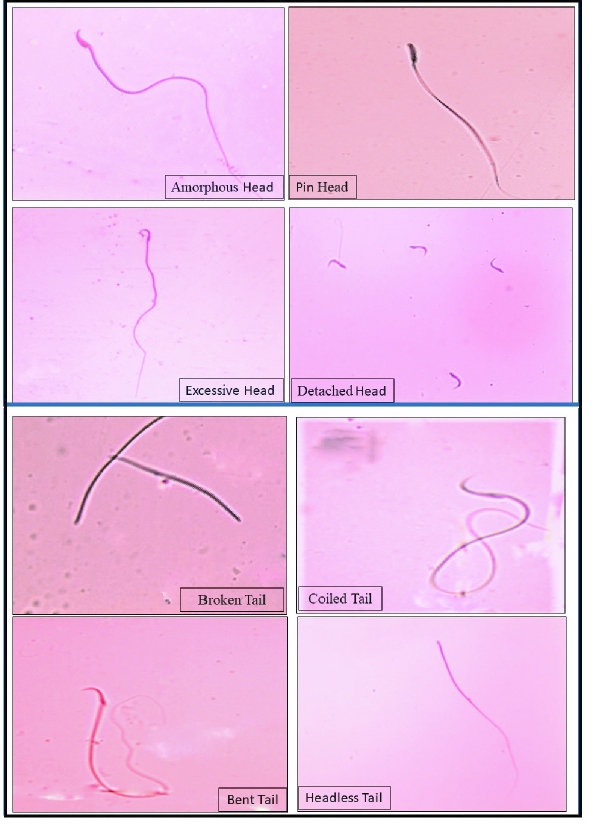
The photomicrographs show the various types of sperm abnormalities observed (eosin stain, 400x).

The results of sperm abnormalities are summarized in (Tables V and VI) which shows the various types of sperm head and tail abnormalities. The results show a significant increase in sperm abnormality with various head and tail abnormalities in the D-gal group, indicating the deleterious effect of D-gal. The TP-treated groups (Post-TP, Comb-TP) showed lower sperm abnormalities when compared to D-gal. Similar trends were observed for SB treated groups (Post-SB, Comb-SB), where the lower sperm head and tail abnormalities were observed as compared to the D-gal group. The microphotographs of the various sperm head and tail abnormalities are shown in (Figure 1).

### Hormone analysis 

#### Serum T level

**Figure 2:**
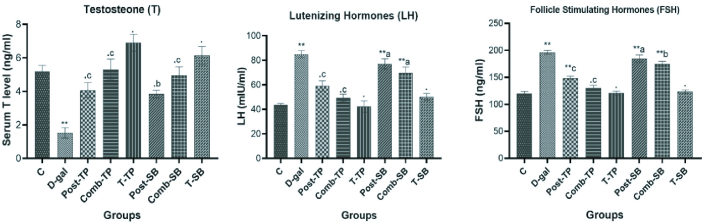
Effects of TP & SB on various reproductive hormones (T, LH, FSH) in D-gal-induced aging male Wistar rats. Values are represented as Mean ± SE for 6 rats in each group. (.) P > 0.05, (**) P < 0.01, compared with control. (a) P > 0.05 (b) P < 0.05, (c) P < 0.01, compared with D-gal.

The D-gal exposed group exhibited a significant (p < 0.001) reduction in serum T level compared to the control group. On the other hand, treatment with TP caused a significant elevation in serum T levels in the treated groups Post-TP (p < 0.001) and Comb-TP (p < 0.001) as compared to the exposure group D-gal. Further, a significant increase in T level was also observed after the administration of SB in both Post-SB (p < 0.001) and Comb-SB (p < 0.001), suggesting that it has a strong influence on testicular steroidogenesis (Figure 2).

#### LH and FSH 

In the D-gal group, the LH and FSH levels in the serum increased significantly (p < 0.001) as compared to the control group. The administration of TP significantly (p < 0.001) reduced the elevated LH and FSH levels in Post-TP and Comb-TP when compared to the exposure group (D-gal). SB-administered groups showed nonsignificant (p > 0.05) decreased LH levels when compared to (D-gal). FSH level was also decreased in Post-SB (p = 0.59) and Comb-SB (p = 0.03) groups as compared to D-gal (Figure 2).

### Steroidogenic enzymes 

**Figure 3:**
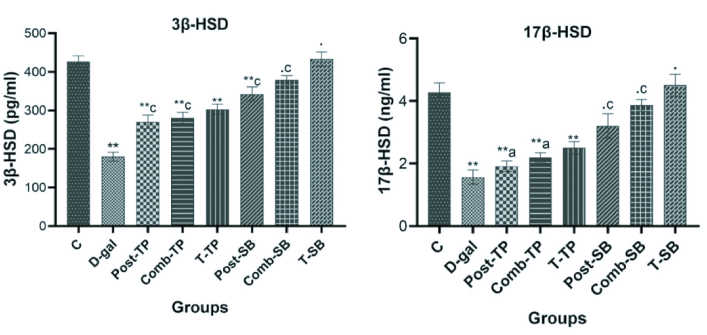
Effects of TP & SB on the steroidogenic enzymes (3β-HSD & 17β-HSD) in D-gal-induced aging male Wistar rats. Values are represented as Mean ± SE for 6 rats in each group. (.) P > 0.05, (**) P < 0.01, compared with control. (a) P > 0.05, (c) P < 0.01, compared with D-gal. 3.5 NF-κB, TNF-α, IL-6 and IL-1β.

The testicular 3β-HSD and 17β-HSD enzyme levels were significantly lowered (p < 0.001) in the exposed group (D-gal) than in the control group. In both the TP-treated groups (Post-TP, Comb-TP) the administration of TP significantly (p < 0.001) increased the 3β-HSD level relative to the exposure group (D-gal); however, this increase was nonsignificant (p > 0.05) in case of 17β-HSD. The administration of SB significantly (p < 0.001) increased the 3β-HSD level and 17β-HSD enzyme levels in both Post-SB and Comb-SB groups when compared to D-gal (Figure 3). The results showed the protective effect of SB and its influence on gonadal steroidogenesis.

The current study's findings reveal a significant (p < 0.001) increase in the serum level of the various inflammatory markers NF-κB, TNF-α, IL-6, and IL-1β in the exposure group (D-gal) in comparison to the control group. TP injection decreased the elevated serum levels of NF-κB, TNF-α, IL-6, and IL-1β in both the TP-treated groups (Post-TP, Comb-TP) compared with the D-gal group. SB inhibited the inflammatory markers by significantly lowering the elevated levels of NF-κB, TNF-α, IL-6, and IL-1β in the (Post-SB and Comb-SB) groups compared to the D-gal group (Table VII).

### Antioxidant enzymes

In testis, the LPO level was significantly increased (p < 0.001) in the D-gal exposed group when compared with the control group. However, the increased LPO in D-gal exposure was significantly (p < 0.001) decreased by TP and SB administration in the Post-TP, Comb-TP, Post-SB, and Comb-SB groups. Several inhibitory responses to the testicular antioxidant status were observed on D-gal exposure.

The D-gal group had significantly (p < 0.001) reduced levels of the antioxidant enzymes SOD, CAT, and nonenzymatic antioxidant GSH when compared to the control group (C). The TP and SB treatments elevated the D-gal mediated reduced SOD, CAT, and GSH in (Post-TP, Comb-TP) and (Post-SB, Comb-SB) groups. The other antioxidant enzymes GR, GPx, and GST activities were also significantly (p < 0.001) reduced in the D-gal group. However, the administration of SB and TP significantly improved the level of these testicular antioxidant enzymes (Table VIII).

### StAR mRNA expression 

**Figure 4:**
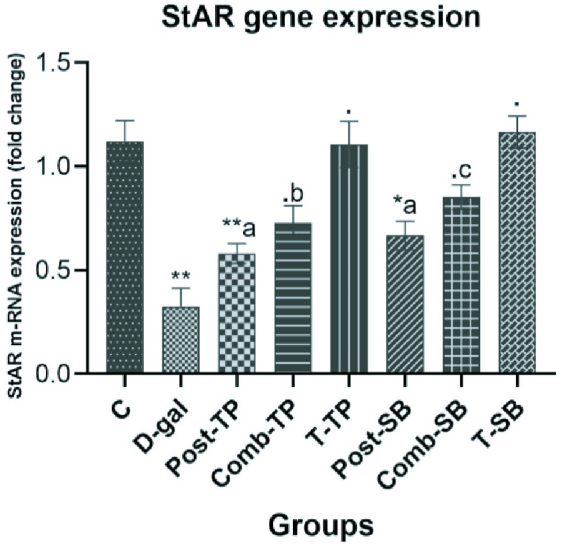
Effects TP and SB on testicular mRNA expression of StAR genes in D-gal induced aging male Wistar rats. Data of the m-RNA expression are expressed as the Mean ± SE (n = 6), normalized to the Rpl-19 m-RNA level, and shown as the fold change (in log2scale) relative to the control mRNA levels. (**.**) P > 0.05, (*) P < 0.05, (**) P < 0.01, compared with control. (a) P > 0.05, (b) P < 0.05, (c) P < 0.01, compared with D-gal.

The RT-PCR results showed that the StAR mRNA expression in the D-gal exposed group was significantly (p < 0.001) downregulated as compared to the control group. The administration of TP upregulated the StAR mRNA expression in both the TP-treated groups Post-TP (p = 0.36) and Comb-TP (p = 0.02) when compared with the exposure group (D-gal). SB therapy significantly upregulated the mRNA expression of the StAR protein in both the Post-SB (p = 0.08) and Comb-SB (p < 0.001) groups as compared to the exposure group (D-gal) (Figure 4).

### Histological examination 

**Figure 5:**
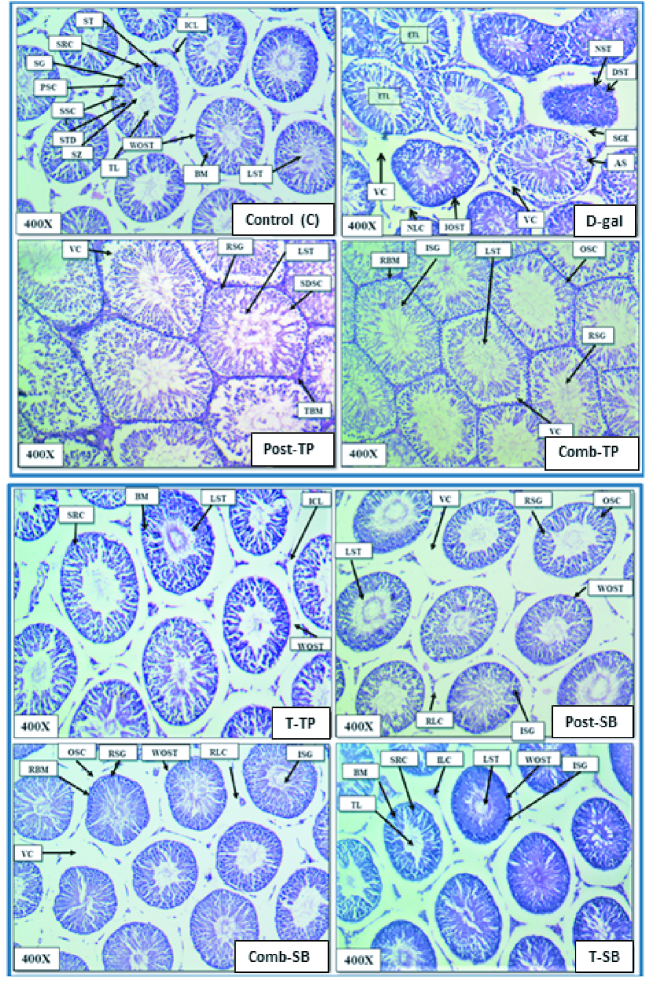
Microphotograph showing histopathological changes in the T.S. of the testis (H&E stained) of male Wistar rats of various treated groups. (Abbreviation used:- ICL: Interstitial cell of Leydig's, ST: Seminiferous tubules, SRC: Sertoli cells, SG: Spermatogonial cells, PSC: Primary spermatocytes, SSC: Secondary spermatocytes, SZ: Spermatozoa, STD: Spermatids, TL: Testis lumen, LST: Lumen filled with spermatids, BM: Basement membrane, WOST: Well organized seminiferous tubules, ETL: Empty testis lumen, VC: Vacuolization of tubules, NST: Narcosis of seminiferous tubules, DST: Degenerated seminiferous tubules, SGE: Sloughed germinal epithelium, AS: Arrested spermatogenesis, NLC: Narcosis of Leydig cells, IOST: Irregular outline of seminiferous tubules, RSG: Recovery in spermatogonia, SDSC: Slightly disorganized Sertoli cell, OSC: Organized Sertoli cells, TBM: Thick basement membrane, RBM: Recovery of the basement membrane, ISG: Increased spermatogonia, RLC: Recovery of Leydig cells).

The histological examination of the testes of different groups stained with H&E at 400x magnification is shown in (Figure 5). The testicular micrograph of the control group exhibited the normal histology of seminiferous tubules, having normal intact outline epithelium, normal interstitial cells of Leydig's, Sertoli cells, spermatogonia, primary spermatocytes, secondary spermatocytes, spermatids and spermatozoa.

The micrograph also shows that the testis lumen is filled with sperms, showing a normal spermatogenesis cycle. The micrograph of the D-gal exposed group showed distorted seminiferous tubules with irregular outline epithelium of seminiferous tubules, narcosis of tubules (NST), vacuolization (VC), sloughed germinal epithelium with an empty testis lumen with decreased number of sperm cells. The TP-treated groups Post-TP, and Comb-TP revealed moderate damage and mild germ-cell degeneration, showing that TP partially restored the testicular damage caused by D-gal. The Post-SB and Comb-SB groups showed remarkable improvement in structural alteration induced by D-gal. The combination groups Comb-TP and Comb-SB showed better recovery in testicular structure than the post treatment groups Post-TP and Post-SB.

**Table 3:** Effects of TP and SB on Bwt and accessory reproductive organs weight in D-gal-induced aging male Wistar rats


**Groups**	**Body weight (g)**	**Testis weight (g)**	**Cauda's weight (g)**	**Prostate weight (g)**	**Seminal vesicle weight (g)**
**C**	229.5 ± 5.812	1.412 ± 0.057	0.34 ± 0.02	0.28 ± 0.01	0.414 ± 0.025
**D-gal**	182 ± 10.29 ${}^{***}$	1.042 ± 0.048 ${}^{**}$ *	0.22 ± 0.02 ${}^{*}$	0.23 ± 0.01 ${}^{*}$	0.292 ± 0.016 ${}^{*}$
**Post-TP**	222 ± 10.46 ${}^{*}$ ${}^{\text{b}}$	1.340 ± 0.069 ${}^{*}$ ${}^{\text{c}}$	0.41 ± 0.03 ${}^{*}$ ${}^{\text{c}}$	0.61 ± 0.02 ${}^{***\text{b}}$	0.604 ± 0.023 ${}^{**\text{b}}$
**Comb-TP**	209.5 ± 6.37 ${}^{*}$ ${}^{\text{a}}$	1.293 ± 0.046 ${}^{*}$ ${}^{\text{b}}$	0.45 ± 0.03 ${}^{*}$ ${}^{\text{a}}$	0.61 ± 0.04 ${}^{***\text{b}}$	0.707 ± 0.032 ${}^{***\text{b}}$
**T-TP**	231.33 ± 6.16 ${}^{*}$	1.301 ± 0.026 ${}^{*}$	0.48 ± 0.04 ${}^{**}$	0.40 ± 0.02 ${}^{**}$	1.164 ± 0.079 ${}^{**}$ *
**Post-SB**	212.83 ± 10.47 ${}^{*\text{a}}$	1.449 ± 0.048 ${}^{*}$ ${}^{\text{c}}$	0.30 ± 0.03 ${}^{*}$ ${}^{\text{a}}$	0.31 ± 0.02 ${}^{*}$ ${}^{\text{a}}$	0.385 ± 0.047 ${}^{*}$ ${}^{\text{a}}$
**Comb-SB**	225 ± 6.72 ${}^{*\text{b}}$	1.323 ± 0.019 ${}^{*}$ ${}^{\text{c}}$	0.34 ± 0.02 ${}^{*}$ ${}^{\text{a}}$	0.25 ± 0.01 ${}^{*}$ ${}^{\text{a}}$	0.487 ± 0.041 ${}^{*\text{a}}$
**T-SB**	233.33 ± 7.6*	1.339 ± 0.052 ${}^{*}$	0.37 ± 0.01 ${}^{*}$	0.36 ± 0.04 ${}^{*}$	0.505 ± 0.020 ${}^{*}$
Values are represented as Mean ± SE. One-way ANOVA followed by Tukey's post hoc test. (*) P > 0.05, (**) P < 0.05, (***) P < 0.01, compared with control. (a) P > 0.05, (b) P < 0.05, (c) P < 0.01, compared with D-gal. C: Control, D-gal: Exposure of D-galactose, Post-TP: Post treatment of testosterone propionate, Comb-TP: Combination of D-galactose and testosterone propionate, T-TP: Treatment of testosterone propionate, Post-SB: Post treatment of *Swarna Bhasma*, Comb-SB: Combination of D-galactose and *Swarna Bhasma*, T-SB: Treatment of *Swarna Bhasma*					

**Table 4:** Effects of TP & SB on sperm counts and sperm motility in D-gal-induced aging male Wistar rats


**Groups**	**Sperm counts (in millions)**	**Sperm motility (%)**
**C**	33.67 ± 1.17	79 ± 4.85
**D-gal**	19.33 ± 1.00 ${}^{***}$	57.83 ± 4.48 ${}^{***}$
**Post-TP**	23.42 ± 1.87 ${}^{**\text{a}}$	62.83 ± 3.95 ${}^{**\text{a}}$
**Comb-TP**	26.00 ± 2.05 ${}^{\text{a}}$	68.67 ± 2.22 ${}^{\text{a}}$
**T-TP**	36.08 ± 2.18 ${}^{*}$	83.16 ± 1.07 ${}^{*}$
**Post-SB**	25.60 ± 2.67 ${}^{\text{a}}$	64.83 ± 3.58 ${}^{\text{a}}$
**Comb-SB**	28.17 ± 1.01 ${}^{\text{b}}$	70.33 ± 2.42 ${}^{\text{a}}$
**T-SB**	37.75 ± 2.14 ${}^{*}$	79.50 ± 2.64 ${}^{*}$
Values are represented as Mean ± SE. (*) P > 0.05, (**) P < 0.05, (***) P < 0.01, compared with Control. (a) P > 0.05, (b) P < 0.05, compared with D-gal. One-way ANOVA followed by Tukey's post hoc test. C: Control, D-gal: D-galactose, TP: Testosterone propionate, Comb: Combination, Post T: Post Treatment, SB: *Swarna Bhasma*		

**Table 5:** Effects of TP and SB on various sperm head abnormalities (%) in D-gal-induced aging male Wistar rats


**Groups**	**Excessive hook (%)**	**Amorphous hook (%)**	**Pin head (%)**	**Detached head (%)**
**C**	0.58 ± 0.24	0.33 ± 0.25	0.67 ± 0.17	2.25 ± 0.31
**D-gal**	2.25 ± 0.21 ${}^{*}$	1.08 ± 0.15 ${}^{*}$	1.41 ± 0.08 ${}^{*}$	6.16 ± 0.46 ${}^{***}$
**Post-TP**	1.08 ± 0.64 ${}^{\text{a}}$	0.92 ± 0.35 ${}^{\text{a}}$	1.08 ± 0.35 ${}^{\text{a}}$	2.92 ± 0.54 ${}^{\text{c}}$
**Comb-TP**	1.17 ± 0.17 ${}^{\text{a}}$	0.83 ± 0.28 ${}^{\text{a}}$	1.17 ± 0.25 ${}^{\text{a}}$	3.25 ± 0.31 ${}^{\text{c}}$
**T-TP**	0.75 ± 0.13 ${}^{*}$	0.38 ± 0.15 ${}^{*}$	0.75 ± 0.25 ${}^{*}$	2.08 ± 0.30 ${}^{*}$
**Post-SB**	1.25 ± 0.11 ${}^{\text{a}}$	0.83 ± 0.28 ${}^{\text{a}}$	1.01 ± 0.21 ${}^{\text{a}}$	2.75 ± 0.44 ${}^{\text{c}}$
**Comb-SB**	1.05 ± 0.11 ${}^{\text{a}}$	0.75 ± 0.17 ${}^{\text{a}}$	0.92 ± 0.28 ${}^{\text{a}}$	2.50 ± 0.48 ${}^{\text{c}}$
**T-SB**	0.58 ± 0.15 ${}^{*}$	0.42 ± 0.25 ${}^{*}$	0.58 ± 0.15 ${}^{*}$	2.12 ± 0.47 ${}^{*}$
Values are represented as Mean ± SE for 6 rats in each group (*) P > 0.05, (**) P < 0.01, compared with control. (a) P > 0.05, (b) P < 0.01, (c) P < 0.01, compared with D-gal. One-way ANOVA followed by Tukey's post hoc test*.* C: Control, D-gal: D-galactose, TP: Testosterone propionate, Comb: Combination, Post T: Post Treatment, SB: *Swarna Bhasma*				

**Table 6:** Effects of TP and SB on various sperm tail abnormalities (%) in D-gal-induced aging male Wistar rats


**Groups**	**Head less tail (%)**	**Broken tail (%)**	**Coiled tail (%)**	**Bent tail (%)**
**C**	1.83 ± 0.28	1.66 ± 0.21	1.75 ± 0.11	1.42 ± 0.15
**D-gal**	4.42 ± 0.58 ${}^{***}$	5.17 ± 0.51 ${}^{***}$	4 ± 0.52 ${}^{***}$	3.75 ± 0.38 ${}^{***}$
**Post-TP**	3.42 ± 0.35 ${}^{\text{a}}$	2.5 ± 0.29 ${}^{\text{c}}$	2.08 ± 0.30 ${}^{\text{c}}$	2.08 ± 0.30 ${}^{\text{c}}$
**Comb-TP**	2.75 ± 0.31 ${}^{**\text{b}}$	2.67 ± 0.28 ${}^{\text{c}}$	1.92 ± 0.15 ${}^{\text{c}}$	1.83 ± 0.33 ${}^{\text{c}}$
**T-TP**	1.80 ± 0.28 ${}^{*}$	1.58 ± 0.15 ${}^{*}$	1.42 ± 0.30 ${}^{*}$	1.55 ± 0.31 ${}^{*}$
**Post-SB**	2.5 ± 0.22 ${}^{\text{c}}$	1.75 ± 0.17 ${}^{\text{c}}$	2.0 ± 0.15 ${}^{\text{c}}$	1.92 ± 0.37 ${}^{\text{c}}$
**Comb-SB**	2.25 ± 0.28 ${}^{\text{c}}$	2.17 ± 0.46 ${}^{\text{c}}$	1.75 ± 0.34 ${}^{\text{c}}$	1.75 ± 0.30 ${}^{\text{c}}$
**T-SB**	1.75 ± 0.11 ${}^{*}$	1.17 ± 0.21 ${}^{*}$	1.92 ± 0.20 ${}^{*}$	1 ± 0.13 ${}^{*}$
Values are represented as Mean ± SE for 6 rats in each group. (*) P > 0.05, (**) P < 0.05, (***) P < 0.01, compared with control. (a) P > 0.05, (b) P < 0.05, (c) P < 0.01, compared with D-gal. One-way ANOVA followed by Tukey's post hoc test. C: Control, D-gal: D-galactose, TP: Testosterone propionate, Comb: Combination, Post T: Post Treatment, SB: *Swarna Bhasma*				

**Table 7:** Effects of TP & SB on various inflammatory markers (NF-κB, TNF-α, IL-6, and IL-1β) in serum of D-gal-induced aging male Wistar rats


**Groups**	**NF-kβ (ng/ml)**	**TNF-α (ng/ml)**	**IL-1β (pg/ml)**	**IL-6 (pg/ml)**
**C**	39.7 ± 3.02	50.04 ± 3.95	305.73 ± 17.39	10.15 ± 1.07
**D-gal**	79.18 ± 3.29 ${}^{***}$	90.78 ± 3.21 ${}^{***}$	460.31 ± 23.78 ${}^{***}$	17.51 ± 1.72 ${}^{***}$
**Post-TP**	53.05 ± 3.53 ${}^{\text{c}}$	80.07 ± 3.56 ${}^{***\text{a}}$	425.87 ± 24.77 ${}^{***\text{a}}$	13.03 ± 0.81 ${}^{\text{a}}$
**Comb-TP**	45.25 ± 2.21 ${}^{\text{c}}$	66.93 ± 3.65 ${}^{**\text{c}}$	388.58 ± 19.75 ${}^{\text{a}}$	14.58 ± 1.52 ${}^{\text{a}}$
**T-TP**	41.43 ± 3.04 ${}^{*}$	54.77 ± 2.41 ${}^{*}$	319.6 ± 21.32 ${}^{*}$	8.27 ± 0.72 ${}^{*}$
**Post-SB**	51.07 ± 3.98 ${}^{\text{c}}$	75.38 ± 4.19 ${}^{***\text{a}}$	419.53 ± 11.42 ${}^{***\text{a}}$	12.02 ± 0.6 ${}^{\text{b}}$
**Comb-SB**	42.26 ± 2.57 ${}^{\text{c}}$	63.08 ± 4.25 ${}^{\text{c}}$	349.48 ± 12.42 ${}^{\text{c}}$	12.61 ± 1.19 ${}^{\text{a}}$
**T-SB**	39.37 ± 2.88 ${}^{*}$	59.04 ± 3.42 ${}^{*}$	308.28 ± 16.79 ${}^{*}$	10.16 ± 0.67 ${}^{*}$
Values are represented as Mean ± SE for 6 rats in each group. (*) P > 0.05, (**) P < 0.05, (***) P < 0.01, compared with Control. (a) P > 0.05, (b) P < 0.05, (c) P < 0.01, compared with D-gal. One-way ANOVA followed by Tukey's post hoc test. NF-kβ: Nuclear factor kappa-light-chain-enhancer, TNF-α: Tumor necrosis factor alpha, IL-1β: Interleukin-1beta, IL-6: Interleukin-6, C: Control, D-gal: D-galactose, TP: Testosterone propionate, Comb: Combination, Post T: Post Treatment, SB: *Swarna Bhasma*				

**Table 8:** Effects of TP and SB on testicular antioxidant enzymes in D-gal induced aging male Wistar rats


**Groups**	**LPO (nmole MDA/hr/gram/tissue)**	**SOD (Unit/mg protein)**	**CAT (µ mole of H ${}_{2}$ O ${}_{2}$ /Unit/mg protein)**	**GSH (µmole/g tissue)**	**GR (nM/min/mg protein)**	**GST Activity (µ mole CDNB/min/mg protein)**	**GPx (µ mole/min/mg protein)**
**C**	3.11 ± 0.161	20.33 ± 1.44	168.94 ± 5.63	6.06 ± 0.76	179.14 ± 3.99	20.33 ± 1.44	11.36 ± 0.61
**D-gal**	9.40 ± 0.43 ${}^{***}$	8.66 ± 1.0 ${}^{***}$	67.06 ± 3.77 ${}^{***}$	2.48 ± 0.45 ${}^{***}$	84.75 ± 3.27 ${}^{***}$	8.66 ± 1.09 ${}^{***}$	4.75 ± 0.76 ${}^{***}$
**Post-TP**	6.74 ± 0.34 ${}^{***\text{c}}$	11.08 ± 0.96 ${}^{***\text{a}}$	100.07 ± 2.95 ${}^{***\text{c}}$	3.66 ± 0.98 ${}^{\text{a}}$	95.85 ± 2.31 ${}^{***\text{a}}$	11.08 ± 0.96 ${}^{***\text{a}}$	6.08 ± 0.91 ${}^{***\text{a}}$
**Comb-TP**	5.79 ± 0.40 ${}^{***\text{c}}$	13.24 ± 1.54 ${}^{***\text{a}}$	105.95 ± 4.08 ${}^{***\text{c}}$	4.7 ± 0.54 ${}^{\text{a}}$	109.16 ± 2.81 ${}^{***\text{b}}$	13.24 ± 1.54 ${}^{***\text{a}}$	6.86 ± 0.8 ${}^{**\text{a}}$
**T-TP**	4.53 ± 0.20 ${}^{*}$	15.02 ± 1.81 ${}^{*}$	170.19 ± 5.23 ${}^{*}$	5.47 ± 0.5 ${}^{*}$	160.12 ± 5.9 ${}^{*}$	20.82 ± 2.29 ${}^{*}$	8.94 ± 0.95 ${}^{*}$
**Post-SB**	5.33 ± 0.44 ${}^{\text{c}}$	15.16 ± 0.85 ${}^{\text{b}}$	126.16 ± 4.07 ${}^{***\text{c}}$	4.13 ± 0.63 ${}^{\text{a}}$	104.5 ± 5.53 ${}^{***\text{a}}$	15.16 ± 0.85 ${}^{\text{bad hbox}}$	6.9 ± 0.69 ${}^{**\text{a}}$
**Comb-SB**	4.17 ± 0.59 ${}^{\text{c}}$	16.53 ± 1.01 ${}^{\text{c}}$	131.32 ± 4.15 ${}^{***\text{c}}$	5.35 ± 0.15 ${}^{\text{b}}$	123.08 ± 7.66 ${}^{***\text{c}}$	16.53 ± 1.01 ${}^{\text{c}}$	7.93 ± 1.12 ${}^{\text{a}}$
**T-SB**	3.08 ± 0.35 ${}^{*}$	19.02 ± 1.4 ${}^{*}$	172.58 ± 4.92 ${}^{*}$	5.52 ± 0.3 ${}^{*}$	170.76 ± 7.27 ${}^{*}$	23.18 ± 1.59 ${}^{*}$	9.95 ± 0.79 ${}^{*}$
Values are represented as Mean ± SE for 6 rats in each group. (*) P > 0.05, (**) P < 0.05, (***) P < 0.01, compared with Control. (a) P > 0.05, (b) P < 0.05, (c) P < 0.01, compared with D-gal. One-way ANOVA followed by Tukey's post hoc test. LPO: Lipid peroxidation, SOD: Superoxide dismutase, CAT: Catalase, GSH: Glutathione reductase, GR: Glutathione reductase, GST: Glutathione-S-transferase, GPx: Glutathione peroxidase, C: Control, D-gal: D-galactose, TP: Testosterone propionate, Comb: Combination, Post T: Post Treatment, SB: *Swarna Bhasma*							

## 4. Discussion

### D-gal

In the present investigation, D-gal exposure resulted in decreased Bwt, sex organs weight, sperm count, and motility with increased sperm abnormalities. The decline in Bwt in D-gal-exposed Wistar rats can be primarily related to the loss of lean tissue, including muscle mass and bone, in aging animals. Similar results have been reported in aging human beings (33). The weight of the testis and accessory sex organs mainly depends on the mass of the differentiated spermatogenic cells. Thus, reduced testicular weight in D-gal exposed animals could be due to a decrease in the number of sperm cells, inhibition of spermatogenesis, and steroidogenic enzyme activities. The observed weight loss of accessory sex organs (cauda epididymis, prostate, seminal vesicles) weights may be due to the reduced bioavailability of sex hormones. A similar finding has also been reported where decreased reproductive organs weight was observed in D-gal-treated mice (34).

Sperm cell analysis is the most effective method for detecting male reproductive disorders. Our results indicated that exposure to D-gal led to a decrease in sperm count as well as an increase in the number of sperms with abnormal morphology. The decline in sperm count may be due to D-gal-induced oxidative stress and decreased androgen hormone production causing germ cell apoptosis. The increased sperm abnormalities were possibly due to increased peroxidative damage to the sperm membrane as it is composed of lipids with high levels of polyunsaturated fatty acids, sphingomyelin, and plasmalogen. Excessive reactive oxygen species (ROS) production may result in loss of membrane and DNA integrity in spermatozoa, thus impairing their fertilization ability. Our results of low-sperm count, motility, and increased sperm abnormalities as the toxic effects of D-gal are concurrent with several earlier reports (1, 4).

Furthermore, a significant decrease in T levels with elevated LH and FSH levels have been observed in D-gal exposed animals. The reduced T production in D-gal might be due to inhibited steroidogenesis owing to oxidative damage to Leydig cells in the testis, as seen in our histological reports. The observed increase in LH and FSH levels on D-gal exposure may be a compensatory feedback response of the hypothalamic and pituitary axis due to the lower T level. But the Leydig cells might become relatively insensitive to increased LH and consequent deficient signal transduction, leading to reduced T synthesis that also characterizes aging Leydig cells. Reduced T levels with increased serum LH and FSH levels in D-gal-induced aging mice has also been reported in earlier studies (35). Comparable trends of decreasing levels of several sex hormones T, dehydroepiandrosterone, with relative increases in LH, FSH, and sex hormone-binding globulin, have also been observed in aging men (33).

These findings suggest that the D-gal animal model for aging seems closer to the human aging of the male reproductive system with T deficiency. Further, D-gal caused a significant decline in steroidogenic enzyme activities (3β-HSD and 17β-HSD) with the downregulation of StAR protein expression. The decrease in the steroidogenic enzyme activities may be possible by two defects along the pathways, LH-stimulated cAMP production and cholesterol transport into the mitochondria via StAR protein, resulting in decreased steroidogenesis. Similar reports of decreased expression of 3β-HSD and 17β-HSD have been reported after D-gal exposure in the rat aging model (1). Downregulation of StAR protein may be due to D-gal mediated oxidative stress, suggesting possible direct inhibitory effects on *StAR* gene expression.

D-gal exposure resulted in a significant increase in various pro-inflammatory markers such as NF-κB, TNF-α, IL-6, and IL-1β. Excessive D-gal accumulation may play a role in enhancing the various inflammatory markers, either directly or via the formation of LPO products. Increased oxidative stress might activate the redox-sensitive transcription factor NF-κB, which plays an important role in inflammatory, immune response, survival, and apoptosis processes. The present finding is in line with the earlier study, who demonstrated a significant increase in the level of TNF-α, IL-1β, IL-6 and other inflammatory factors in the testes of the D-gal exposed Sprague-Dawley male rats (36).

In this study, the antioxidant enzymes activities (SOD, CAT, GPx, GR, GST) and GSH levels decreased with a significant increase in LPO level after D-gal exposure. It is well-documented that the male reproductive organs are particularly susceptible to the harmful effects of ROS. The increased LPO level might be due to D-gal mediated oxidative damage to the testicular membrane because of comparably higher unsaturated fatty acid levels in this tissue than others. The decreased level of first-line antioxidant defense enzymes SOD, CAT, and GPx reflects less testicular mitochondria and microsome capacity to eliminate H ${}_{2}$ O ${}_{2}$ produced by D-gal in oxidative stress. GST, GR, and GSH are the other prime antioxidant enzymes that play a vital role in cellular defense from oxidative stress. The D-gal-induced rat aging model revealed a decline in several enzymatic and non-enzymatic antioxidants which might be due to increased oxidative stress (1). These findings advocate an imbalance of antioxidants with increased free radical production that might be responsible for various observed degenerative changes in the testis. The present study's results agree with the earlier studies that demonstrated decreased antioxidant enzyme activities as the deleterious effect of D-gal. D-gal exposure has also caused various testicular histopathological impairments in this study.

The results suggested that D-gal exerted toxic effects on rat testis through increased oxidative stress, causing various testicular structural damages. Earlier studies have also shown various structural alterations such as damaged seminiferous tubules, decreased epithelium layers, and decreased spermatogenic cells in histopathological investigations of mice exposed to D-gal (37). Changes in male reproductive systems caused by D-gal, such as testicular damage, decreased sperm count, and reduced androgen production with increased serum LH and FSH, are similar to normal aging (2). Based on the present findings, it can be concluded that D-gal exposure caused several alterations in the male reproductive system via increased production of ROS, enhanced inflammation cascade, and reduced androgen production.

### Treatment with TP and SB

In the current study, TP treatment increased the Bwt, testis weight, and other accessory reproductive organs weights. The increase in Bwt may be due to T mediated increase in muscle and bone mass, while an increase in reproductive organs weight can be related to increased androgen availability.

TP therapy resulted in a considerable rise in prostate weight. The increased prostate weight could be the result of androgen receptor-dependent transcription of specific target genes, which leads to the production and secretion of peptide growth factors. Further, a significant increase in sperm count motility with sperm quality was observed after TP treatment. The restoration of spermatogenesis by TP treatment suggests the feasibility of using endocrine regimens to ameliorate the deleterious effects of D-gal on the male reproductive system. It can be hypothesized that increased spermatogenic index (sperm count, motility, and quality) may be due to TP-mediated improvement in antioxidant status, increased T availability in the testis with decreased apoptosis, and increased germ cell protection. Combined treatment of T and estrogen significantly increased the sperm concentration via increasing proliferation and differentiation of germ cells in heat-induced testicular dysfunction rat model (38).

Additionally, the TP treatment also elevated the serum T level and reduced the augmented LH and FSH in D-gal exposed rats. The increase in the T level suggests that the daily exogenous TP treatment was enough to keep plasma T levels constantly high in the D-gal exposed rats. The decrease in LH and FSH levels upon TP treatment might be due to the negative feedback effect of the exogenous treatments on the HPG axis as it inhibits gonadotropin-releasing hormone, thereby preventing the secretion of LH and FSH. Consistent with this study, TP significantly decreased the rate of sperm abnormalities in the epididymis, prevented the reductions in sperm concentration, motility, and antagonized the endosulfan-induced declines in spermatogenous cell and sperm counts in the testes (39).

Additionally, similar reports have been reported where exogenous T protected spermatogenesis due to its antioxidant and anti-inflammatory properties in intact male rats (40). Further, TP also increased the activities of steroidogenic enzymes (3β-HSD and 17β-HSD) in the testis with the upregulation of StAR protein expression. The increase in steroidogenic enzyme activity and upregulation of StAR protein expression might be due to TP's anti-oxidative activity, which protected the mitochondria and ER of Leydig cells from the toxic effects of ROS induced by D-gal.

The TP treatment has been shown to reduce the D-gal-induced elevated levels of pro-inflammatory cytokines viz. TNF-α, IL-1β, IL-6, and redox sensitive transcription factor NF-κB. The TP also ameliorated the D-gal toxicity by decreasing testicular LPO with a significant increase in antioxidant enzymes (SOD, CAT, GST, etc.). The increased antioxidant activities might be responsible for the effective scavenging of ROS, thereby protecting the testicular structure from the toxic effects of D-gal. The antioxidant and anti-inflammatory effects of TP, observed in the current study, might be responsible for its protective effects. Earlier study has reported an ameliorative effect of TP against endosulfan-induced testicular toxicity, with a decrease in testicular LPO and a significant increase in SOD, CAT, and GPx activities (41).

Moreover, the TP treatment also ameliorated the various testicular histopathological alterations caused by D-gal exposure. The cytoprotective activity of TP may be due to increased antioxidant enzyme activity, and protection of the testis from D-gal mediated oxidative damages. Histopathological analysis in previously conducted study exhibited a normal histoarchitecture of testis after TP treatment through amelioration of testicular oxidative stress in rat and mice model (38, 39).

The results suggest that exogenous T treatment may be responsible for improving steroidogenesis and spermatogenesis in aging rat testis. However, long-term TP treatment has been linked with prostate cancer and a potential risk to cardiac health, obstructive sleep apnea, and erythrocytosis (14). A decrease in intratesticular T production has also been reported in long-term therapy of TP, indicating its dependency. Hence, we studied the steroidogenic effect of SB, a natural substance with the capability of enhancing endogenous T production.

SB is an *Ayurvedic* preparation used as sexual function enhancer in traditional Indian *Ayurvedic* medicine (20). In the current investigation, the oral administration of SB significantly increased the mean Bwt, testis weight, and other accessory sex organs weights. Further, treatment of SB also considerably improved sperm counts and motility with a reduction in sperm abnormalities in D-gal exposed rats. The increased spermatogenic activity is related to SB's anti-oxidative and ROS scavenging properties, with greater androgen availability in treated animals. It has been reported that the SB (gold ash) treatment increased the total sperm count and motility percentage in healthy adults and infertile patients (41).

**Figure 6:**
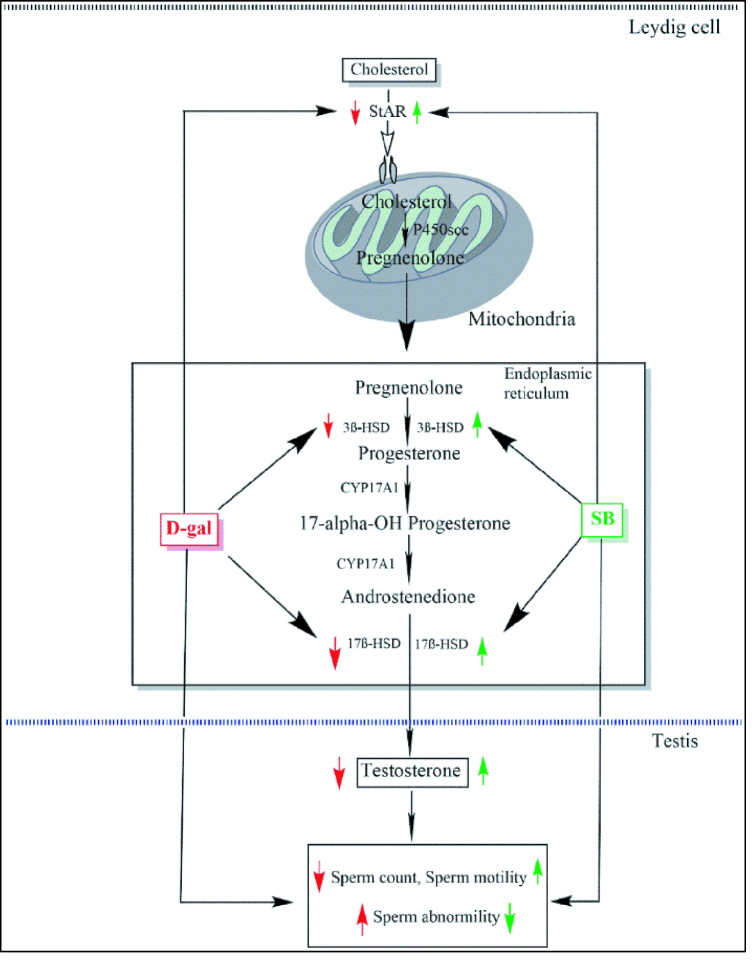
Schematic representation of the ameliorative effects of the SB on D-gal-induced steroidogenic enzymes and spermatogenic alterations.

In the present study, we found that SB significantly increased the serum T level and reduced LH and FSH hormones in D-gal exposed rats. The SB treatment also increased the steroidogenic enzyme activities (3β-HSD, 17β-HSD) with upregulation in StAR protein expression. Gold may be activating the StAR protein expression by activating its gene, through some unknown mechanisms. The increased T level is due to increased steroidogenesis, as evident from increased activities of 3β-HSD and 17β-HSD and upregulated expression of StAR protein (Figure 6).

**Figure 7:**
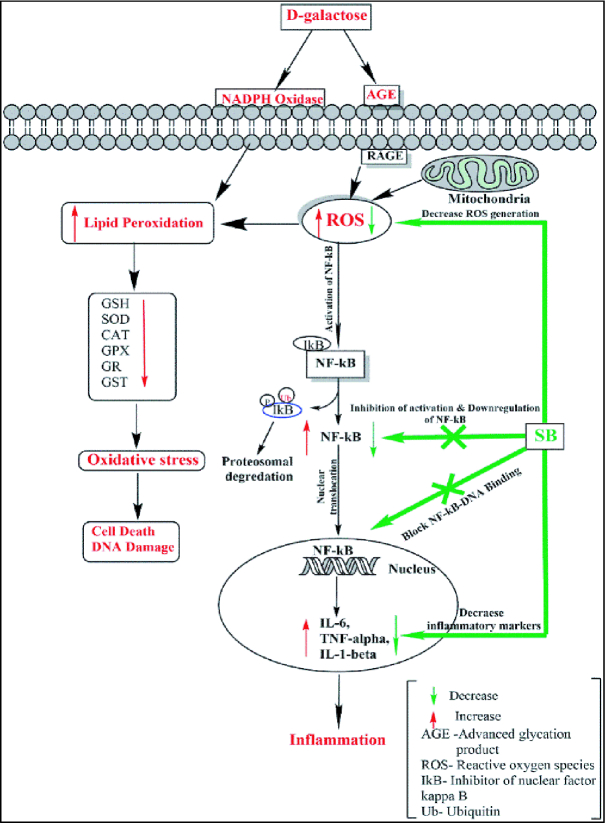
A possible mechanism for a protective effect of SB on D-gal-induced inflammation and testicular antioxidants enzymatic alterations.

The treatment of SB restored the elevated levels of NF-κB, TNF-α, IL-6, and IL-1β upon D-gal exposure toward the level of control. The protective effect of SB treatment may be correlated with its antioxidant, anti-inflammatory activity against the D-gal-induced inflammation cascade. The possible reason for the reduced level of NF-κB in the SB-treated group may be the interaction of gold with the thiol group of the protein responsible for regulating the transcription of the gene controlling cytokine expression. Furthermore, SB effectively protected the testis from D-gal-induced oxidative stress by increasing various antioxidant enzymes (SOD, CAT, GPX, GR, GST) and decreasing the elevated LPO levels at the same time. The antioxidant defence system is the major protective mechanism against oxidative stress that scavenges harmful ROS produced in the testis (Figure 7).

Moreover, in our study, the D-gal testicular histopathological alteration was markedly ameliorated after the SB administration. SB's beneficial effect against D-gal-induced testicular structural alterations can be linked to its anti-oxidative and anti-inflammatory properties. The SB-treated groups (Post-SB, Comb-SB) showed better testicular histoarchitecture than TP treatment (Post-TP, Comb-TP) as seen in our study.

SB is considered as herbo metallic formulation where herbal extracts used in the preparation of SB weren't used for their medicinal benefits but rather for their capacity to chemically reduce the gold metal and mechanically simplify the grinding process (42). In order to understand the composition, the physico-chemical characterization of SB used in the study has been previously characterized by various modern methods like XRD and EDX, which revealed that the major content of SB was gold (97.40%) with a trace amount of Na, Fe, Al, K, Ag, and Ca (43). The repeated incineration cycles during the preparation of SB might result in the breaking of the organic phytochemical molecules in their atomic form. The present study showed that SB has a protective effect against D-gal-induced testicular alterations. SB particles might enter the cellular system and show therapeutic responses through an unknown mechanism. However, a better understanding of the mechanism and pathway involved in the therapeutic action of SB would require further study.

In our study, both the treatment of TP and SB showed a protective effect against the various alterations caused by D-gal. However, previous research showed that testosterone therapy might cause a number of adverse effects, such as symptoms of benign prostatic hypertrophy, liver toxicity, hyperviscosity, erythrocytosis, sleep apnea, or serious heart failure (14).

According to Ayurveda, the ayurvedic metallic formulation is free from any toxic effects. Besides its traditional belief, various in-vitro and in-vivo toxicological studies conducted previously concluded that SB is nontoxic (19). Toxicity study of 90 days was conducted in Wistar rats where a high dose of SB(13.5  mg/kg Bwt) did not cause any alteration in weight and histopathology of organs (liver, kidney) in treated rats (24).

## 5. Conclusion

The study concluded that the D-gal caused several reproductive impairments through increased oxidative stress and decreased T synthesis, affecting sperm count, motility, and morphology. TP and SB reduced oxidative stress, increased sperm count, increased steroidogenesis, and improved testicular histopathology in the D-gal aging model. The findings suggested that maintaining androgen levels through exogenous T treatment may help in sustaining gonadal activity. But the exogenous T treatment uses have increased cause for concern because of the various associated reported toxicities. According to this study, SB maintained the T level endogenously via increased antioxidant activity and restored normal spermatogenesis, and steroidogenesis was altered by D-gal. Further study is needed to find out the various target protein of SB to elucidate its exact mechanism of action. The study concludes that SB may prefer over TP as a therapeutic agent against D-gal-induced testicular toxicity, as it has a long history of usage in India, and a number of studies have established its safety in both humans as well as animal models.

##  Conflict of Interest

The authors declare that there is no conflict of interest.
